# Evaluating the growth and cost–benefit analysis of feeding improved indigenous chicken with diets containing black soldier fly larva meal

**DOI:** 10.3389/finsc.2022.933571

**Published:** 2022-09-05

**Authors:** Mwangi K. Waithaka, Isaac M. Osuga, Lucy W. Kabuage, Sevgan Subramanian, Beatrice Muriithi, Ann M. Wachira, Chrysantus M. Tanga

**Affiliations:** ^1^ International Centre of Insect Physiology and Ecology (icipe), Nairobi, Kenya; ^2^ Department of Animal Sciences, Kenyatta University, Nairobi, Kenya; ^3^ Department of Animal Sciences, Jomo Kenyatta University of Agriculture and Technology, Nairobi, Kenya; ^4^ Non-Ruminant Research Institute (NRI), Kenya Agricultural and Livestock Research Organization (KALRO), Naivasha, Kenya

**Keywords:** insects, alternative protein ingredients, poultry feed, feed intake, cost-effectiveness, improved indigenous chicken, food security

## Abstract

The high cost of feed has been the major hindrance to a hindrance to the growth, sustainability, profitability, and expansion of poultry production. Black soldier fly larva (BSFL) meal is one of the most promising alternative protein sources widely accepted globally. This study evaluated the growth performance of improved indigenous chicken (IIC)-fed diets containing different inclusion levels of BSFL meals. The BSFL meal inclusion rates included 0% (Diet0), 5% (Diet1), 10% (Diet2), 15% (Diet3), and 20% (Diet4) as replacement to the expensive fish meal in chick and grower diets. Our results showed that diet significantly affected the average daily feed intake, feed conversion ratio, and average daily weight gain of the chicks. The average daily weight gain and feed conversion ratio, except average daily feed intake of the growers, was not significantly affected by diets. The gross profit margin, cost–benefit ratio, and return on investment of feeding birds with BSFL meal varied significantly. The highest cost–benefit ratio of 2.12 was recorded for birds fed on Diet4. Our findings demonstrate that insect-based feeds can successfully and cost-effectively replace fish meal up to 20% without compromising the growth performance of the birds. Therefore, BSFL meal could be incorporated as an essential part of poultry feed production for IIC, potentially reducing the total feed cost while maintaining optimal production and reducing the cost of meat and egg products.

## Introduction

The livestock sector continues to experience increasing pressure to meet the rising demand for high-value animal protein. The demand for animal products is expected to double in developing countries by 2030, and poultry meat and eggs are among the most widely consumed high-value animal proteins at the global level (1). According to a report from the Organization for Economic Co-operation and Development (2), poultry products account for approximately 45% of animal protein needs required in the next decade. In Sub-Saharan Africa, the consumption of poultry products continues to expand faster than other meat products, making poultry the fastest-growing agricultural subsectors. This is attributed to rapid population growth, urbanization, and greater purchasing power (3).

According to Wong et al. (4), the largest number of households across the world mainly rears indigenous chicken and, in some cases, crossbred species. In developing countries, about 80% of rural households keep poultry, predominantly raising improved indigenous chicken (IIC), which contributes to over 30% of the total white meat consumed globally (5). The IIC breeds are distributed extensively in Africa compared to other livestock species and represents over 70% of the total chicken population. They play a significant role in income generation and livelihoods improvement, particularly in vulnerable communities with over 80% of smallholder farmers. The chickens are hardy and capable capable of thriving in harsh environmental conditions like droughts and poor husbandry practices (6, 7).

In Kenya, the IIC subsector has been identified as an essential poverty eradication tool for rural households (8) and they account for over 80% of the poultry population (9), making them vital in improving food security for smallholder farmers and diversifying agricultural production (10). According to reports from the United States Agency for International Development (11) and Mengesha (12), the increase in health-conscious consumers, greater purchasing power, and urbanization have contributed to a significant rise in the demand for free-ranged IIC products in the country. Therefore, they are an essential source of affordable poultry products for rural households accounting for about 50% of eggs and meat products (13, 14). Nevertheless, IIC production has continued to be characterized by low productivity despite the presence of many research and development initiatives in the country with focus on improving chicken breeds, development of low-cost high-value feed supplementation, and better management practices (13, 14). Although many studies have worked on locally available and affordable feed resources to address poor nutrition in IIC productivity, adoption and sustainability of these interventions have been poor (13, 15).

The high cost of formulated feeds remains one of the most significant challenges in IIC production since feeds account for roughly 70% of total production costs (6). It is due to the scarcity and high cost of feed resources that the IIC sector has not attained its full potential (16, 17). Therefore, the supply of protein sources, mostly fish meal and soybean meal in the poultry feed industry, continues to decline drastically, thus impeding the growth of smallholder poultry production in Kenya and other developing countries (18). This has pushed many government to search for alternative protein ingredients that can economically supplement conventional protein ingredients used in feed formulation without adverse effects on the health and performance of the birds to address the inadequate supply in the animal feed industry.

There is emerging global interest in the use of insect protein as a potential alternative source to replace expensive conventional major protein sources, particularly fish meal (FM) and soybean meal in animal feeds (19). This is because the crude protein (CP) content of insect meals has been demonstrated to range between 35 and 77, with 33%–36% of lipid content (20–24). Furthermore, insects have been shown to have good balance of amino acids with high levels of digestibility and palatability (235,266). The development of innovative, cost-effective, and environmentally friendly options such as farming of black soldier fly larvae (BSFL) on organic waste and recycling the waste into high-quality nutrient-rich biomass is increasingly being considered as an attractive, viable, and sustainable alternative source of protein (38%–62% CP) to substitute animal- and plant-based sources in animal feeds [20, 18, 27,278,299,30,^25^,332). Several studies have also reported that the crude fat content of BSFL meal ranges between 18% and 42% (33, 344). However, the nutritional profile of BSFL meal has been demonstrated to vary considerably depending on the rearing substrates (26, 355, 366, 377, 388, 399 ).

The use of BSFL meal as an alternative to fish meal or soybean meal in poultry, pig, and fish feeds has been advocated worldwide (27) and provides opportunities from income generation (28, 29). Therefore, BSFL meal could be a valuable and affordable source of protein feed ingredient in IIC diets (30, 31), although research attention is highly limited. Therefore, this study evaluates for the first time the effects of diets containing full-fat BSFL meal at different inclusion levels on the growth, and economic performance of Kenya Agricultural and Livestock Research Organization (KALRO) improved indigenous chicken breed.

## Materials and methods

### Ethical approval

Ethical approval for the study was provided by the Institutional Animal Care and Use Committee (IACUC) of Kenya Agricultural and Livestock Research Organization (KALRO)-Veterinary Science Research Institute (VSRI), approval Code No. KALROVSRI/IACUC019/30082019.

### Experimental facility

The feeding trials were conducted at the KALRO, Non-Ruminant Research Centre, in Naivasha, Nakuru County. The research station is located about 76 km from Nairobi, 71 km from Nakuru, and the GPS coordinates 00 43′ 0.0120″ S and 360 26′ 9.2760″ E, latitude -0.71667, longitude 36.43591, altitude 1915 M.

### Experimental feed formulation

The full-fat black soldier fly larva (BSFL) meal was obtained from the International Centre of Insect Physiology and Ecology (icipe), Nairobi, Kenya. The proximate and amino acid composition of the BSFL meal was analyzed according to the methods described by Chia et al. (32). Based on the nutritional profile of the BSFL meal, the other raw materials were integrated to formulate a diet that meets nutritional requirements of the starter and grower chicken according to the National Research Council (NRC) standards for improved indigenous chicken (IIC). The diets for the starters was constituted to have at least 2,800 kcal ME/kg and 18% crude protein (CP), while that of the grower had 2,550 kcal ME/kg and 15% CP. The diets were designated as 0% BSFL meal (Diet0), 5% BSFL meal (Diet1), 10% BSFL meal (Diet2), 15% BSFL meal (Diet3), and 20% BSFL meal (Diet4) inclusion levels (**Table 1**). The NRC feeding standards were used to estimate the chemical composition of the ingredients before the preparation of the feeding trials diets.

**Table 1:** Ingredients used in the formulation of the experimental diets for improved indigenous starter and grower chickens.

Starter phase						Growers phase					
Ingredients	Diet_0_	Diet_1_	Diet_2_	Diet_3_	Diet_4_	Ingredients	Diet_0_	Diet_1_	Diet_2_	Diet_3_	Diet_4_
Maize Grain	58	55.75	56.75	52.75	50.75	Maize germ	46	39	41	49	51
Pollard	1	2	2	4	6	Rice polish	17	27	29	19.5	10
Wheat Bran	17.8	19	18	20	20	Maize Grain	19	15	12	13	18
Sunflower meal	2	2	2	2	2	Cotton seed cake	2	2	1	0	0
Fish meal	20	15	10	5	0	Sunflower meal	4	4	3	0.5	0
Black Soldier Fly larvae	0	5	10	15	20	Fish meal	10	6	2	1	0
Limestone	0.5	0.5	0.5	0.5	0.5	Black Soldier Fly larvae	0	5	10	15	20
Di-calcium Phosphate	0.25	0.25	0.25	0.25	0.25	Limestone	0.5	0.5	0.5	0.5	0.5
Vitamin/Mineral Premix^1^	0.25	0.25	0.25	0.25	0.25	Di-calcium Phosphate	0.5	0.5	0.5	0.5	0.5
Common Salt	0.25	0.25	0.25	0.25	0.25	Vitamin/Mineral Premix^1^	0.5	0.5	0.5	0.5	0.75
						Common Salt	0.5	0.5	0.5	0.5	0.5

^1^Vitamin and mineral premix provided the following per kg of diet: vitamin A, 11500 IU; cholecalciferol, 2100 IU; vitamin E (from dl-tocopherylacetate), 22 IU; vitamin B12, 0.60 mg; riboflavin, 4.4 mg; nicotinamide, 40 mg; calcium pantothenate, 35 mg; menadione (from menadione dimethyl-pyrimidinol), 1.50 mg; folic acid, 0.80 mg; thiamine, 3 mg; pyridoxine, 10 mg; biotin, 1 mg; choline chloride, 560 mg; ethoxyquin, 125 mg; Mn (from MnSO4·H2O), 65 mg; Zn (from ZnO),55 mg; Fe (from FeSO4·7H2O), 50 mg; Cu (from CuSO4·5H2O), 8 mg; I (from Ca(IO3)2·H2O), 1.8 mg; Se, 0.30 mg; Co (from Co2O3), 0.20 mg; Mo,0.16 mg, B, B1, B2, B3, and B4 as experimental diets, BSFL -Black Soldier Fly Meal.

### Experimental birds, housing, and feeding trials

Three hundred and fifteen (315) mixed-sex 1-day-old KALRO-improved indigenous (called Kienyeji in local language) chickens were sourced from the KALRO Naivasha station. They were placed in a round deep litter brooder prepared at the poultry house, which was fitted with infrared bulbs (250 W) to provide heat during the brooding period. The birds were kept in the brooder for 7 days to acclimatize. Thereafter, the birds were weighed and distributed randomly to the 45 cages (experimental units). A round 4-l drinker and plastic tube feeder measuring 0.73-m length by 0.26-m width by 0.48-m height was provided for each experimental unit.

The 63 birds in each experimental units were randomly assigned to one of the five dietary treatments. For the starter phase, the experimental starter diet was provided ad libitum for a period of 8 weeks. Thereafter, the experimental birds were provided a grower diet between the 9th and 18th weeks of age, which comprises the growing phase. Standard health and biosecurity measures were observed to forestall any disease outbreak. All birds were kept under similar conditions and allowed ad libitum access to feed and water throughout the experiment. Each experimental setup was replicated nine times.

At the commencement of the experiments, diets designated for the starter and grower chickens were subjected to proximate analysis to determine the crude protein, fat, crude fiber, and ash contents using the standard methods outlined by AOAC (2012) (33). Feed intake, body weight gain, and survival rates were additional parameters assessed. The birds were weighed after 7 days of acclimatization and then every other week throughout the entire experiments. Birds in each experimental unit were weighed together in a plastic bucket. Feed was weighed and allocated to each experimental unit at the beginning of the 2nd week and increased gradually based on the consumption rate of the growing birds. Experimental birds were allowed ad libitum access to feed and water throughout the experiment. Feed offered to the birds and unconsumed portions were weighed daily using a digital platform weighing scale (XK3190-A12, >300 kg, Gromy Scale Co., Ltd., Hangzhou, China) to calculate the average daily feed intake (ADFI). Total body weight gain and feed consumed were used to calculate the feed conversion ratio (FCR) for each dietary treatment according to the method described by Sumbule et al. (34).

### Cost–benefit analysis of birds fed on different diets with BSFL meals

The key parameters, which included cost–benefit analysis (CBA) and return on investment (RoI) (35), were used to evaluate the economic implication of replacing fish meal in chicken diets with BSFL meal. The cost–benefit ratio (CBR), as an indicator in CBA, was used to summarize the economic value of replacing fish meal with BSFL meal in the diets. Feed costs were calculated from the ingredient prices based on quantities of each item incorporated in the dietary treatments. A CBR value greater than 1 suggests that the benefits of the production exceeded the production costs and vice versa. RoI is a measure of gain/loss generated from an investment relative to the money invested. The higher the RoI value, the better the returns of the project under consideration (35). The gross profit, gross profit margin, cost–benefit ratio (CBR), and return on investment (RoI) were used to determine the economic performance.

The following formulas were used.


$$\begin{array}{c} \text{Gross profit}=\;\text{Sale of bird}-\text{Total Production Cost}\\ \text{Gross profit margin}=(\text{Sale of bird}-\text{Total Production Cost})÷\;\text{Sales of bird}\\ \text{CBR}=\text{Total Production Cost}÷\text{Sale of bird}\\ \text{RoI}=(\text{Gross Profit}\;÷\mathrm{Total Production Cost})\times 100\% \end{array}$$

### Statistical analysis

The data analysis was done using Statistical Analysis System (SAS, version 9.1). Data were subjected to a one-way analysis of variance (ANOVA) to determine the effect of the different diets on performance parameters. Bon-Tukey was applied to differentiate the statistically different means at a p< 0.05 level of significance. The cages represented the experimental units.

## Results and discussion

### Composition of the BSFL meal for feed formulation

The CP and crude fat values of BSFL meal were 43.2 and 29.4%, respectively (**Table 2**). The results obtained in this study are in agreement with those reported by Cummins et al. (36), who reported that BSFL meal contains a high level of protein, with the amino acid profile similar to fish meal and other nutrients that make it a well-balanced feed. The crude fat content of BSFL meal in the present study was higher than the fat contents of fish meal and BSFL meal as reported by Barroso et al. (37) where values ranged 15.6%–18.0%, but it was similar to the values observed by Zulkifli et al. (38) that ranged between 26% and 38%. Interestingly, previous documented information revealed that the yield quantity and quality of BSFL fat depend on the stage of development of the insect and also the processing method (39). The developmental stages of the BSFL can be one of the factors that affect the lipid quantity. According to several authors, high lipid levels can be observed to increase toward the 5th-instar larval stages of the insect due to the metabolic turnover that takes place during the process of metamorphosis (40).

**Table 2:** Proximate and amino acid composition of BSFL meal for feed formulation.

Parameter	Proximate composition of BSFL meal	Amino acid composition (% DM)			
		Essential amino acids	Percentage (%) on dry matter basis	Nonessential amino acids	Percentage (%) on dry matter basis
Dry matter	97.0	Arginine	2.1	Serine	1.8
Crude protein	43.9	Histidine	1.4	Proline	2.4
Crude fat	29.4	Isoleucine	1.8	Alanine	2.6
Crude fiber	21.3	Leucine	2.8	Aspartic acid	3.9
Ash	13.2	Lysine	2.8	Cystine	0.4
		Methionine	0.8	Glycine	2.5
		Phenylalanine	1.6	Glutamic acid	4.6
		Threonine	1.6		
		Valine	2.5		

The ash content of BSFL meal was higher than that observed by Zulkifli et al. (38) and Barroso et al. (37). The fiber content recorded was 21.3%, which is 2.4-fold higher compared to that reported by Zulkifli et al. (38). Crude fiber is a direct estimate of the amount of chitin present in the BSFL meal given that this polysaccharide is the most common form of fiber in insects (29). The crude fiber content in insects depends on the developmental life stage within the life cycle. These results are in agreement with those reported by Kramer and Koga (41), who found that as the larvae progress toward the pre-pupa stage and eventually the pupation stage, chitin content starts to increase significantly. Similarly, Soetemans et al. (42) have also observed this progressive change in chitin quantity and quality in BSFL meal, which will obviously influence the digestibility of the nutrients in animal feed. This explains why Fines and Holt (43) have always emphasized the importance of optimizing the amount of chitin in the feed according to chitinolytic activity in the gut of every given animal category and their ability to digest this substance in deciding the rate of inclusion.

The amino acid composition of BSFL meal in the present study was similar to that reported by Cummins et al. (36) and Zulkifli et al. (38). Leucine, lysine, arginine, and valine were among the highest in essential amino acids in BSFL meal, which is in line with the results demonstrated by St-Hilaire et al. (44). The values of the non-essential amino acids obtained were also comparable to the values presented by St-Hilaire et al. (44). Our findings are consistent with the amino acid profile of three different sources of BSFL meals, which are quite similar to fish meal (45) known as the protein with the best amino acid profile for both human and animal nutrition. Similarly, Barroso et al. (37) further supported our observations and reported that the amino acid profiles of *H. illucens* and other Dipteran larvae like house fly (Musca domestica) were better sources than the soybean meal, which could be used as a suitable replacement of fish meal in animal feed formulation.

Currently, the use of BSFL meal is receiving more attention in the poultry feed industry as an effort to reduce dependency on fish meal or soya bean for protein and oil. In this study, the CP content of the starter diet varied considerably across the diet types with Diet0 containing fish meal recording the highest CP value of 20.8%. The CP of the four poultry diets with BSFL meal had values ranging between 14.2% and 17.4%. For the chicken grower diets, the CP values ranged between 11.3% and 13.2% (**Table 3**).

**Table 3:** Chemical composition of starter and grower chicken diets.

Starter diets							Grower diets					
Parameter	Diet_0_	Diet_1_	Diet_2_	Diet_3_	Diet_4_	Parameter	Diet_0_	Diet_1_	Diet_2_	Diet_3_	Diet_4_
Dry matter	89.8	90.5	91.2	92.0	91.2	Dry matter	94.3	95.3	94.8	93.8	94.7
Crude protein	20.8	14.2	17.0	16.3	17.4	Crude protein	13.1	12.1	13.2	11.3	13.1
Crude fat	3.6	3.8	9.8	11.0	6.5	Crude fat	4.4	4.4	11.7	14.4	10.0
Crude fiber	3.6	3.9	4.1	4.5	4.8	Crude fiber	5.3	5.1	5.2	5.5	5.7
Ash	7.2	14.5	10.7	9.5	5.3	Ash	8.0	12.3	12.5	8.2	8.2

This significant variability in the CP content of the various diet types can be attributed to the processing and inability of the full-fat BSFL meal to mix properly during the compounded feed formulation process. This is a shortcoming in the present study that needs further research attention for effective feed formulations. However, no studies are currently available on these aspects for improved indigenous chicken feeds integrated with BSFL meal. In relation to the nutritive profile, BSFL meal has been reported to contain large amounts of lipids, which might present an extreme challenge during mixing with other feed ingredients (46). Thus, increasing inclusion levels may negatively affect the consistency of the finished product and the feed conversion ratio of the birds (46). This suggests that low inclusion levels (50 or 100 g/kg) or the use of defatted BSFL meal may be a more suitable option. On the other hand, there is a lack of scientific information about the impact of the use of defatted BSFL meal on poultry feed formulation and quality. This is further supported by Zheng et al. (47) who reported that the defatting process results in insect meals with larger protein values and reduces the risk of lipid oxidation, allowing for a longer shelf life of the product.

There was a significant effect on the feed intake, final body weight, and feed conversion ratio when the starter chicken were fed a diet with a full-fat BSFL meal (**Table 4**). Birds fed Diet1 had the highest ADFI, although no significant differences were observed between Diet1, Diet2, and Diet3. Starter chicken provided Diet0 showed a significantly higher average daily weight gain (ADWG) when compared to other dietary treatment. The FCR among starter birds fed Diet0 and Diet2 as well as Diet3 and Diet4 was not significantly different (**Table 4**). However, the FCR of the grower birds did not vary across the various diets. These results of FCR are in agreement with those reported by Dabbou et al. (48) and de Souza et al. (49) for broiler chicken and Al-Qazzaz et al. (50) for layer chickens. Contrarily, Mat et al. (51) observed a lower FCR following higher inclusion levels of defatted BSFL meal in broiler starter diets. The BSFL meal-based feeds showed no significant effect on the final body weight and FCR when birds were fed the various diet types during the growing phase. There was a significant treatment effect on feed intake for grower chicken fed the various diets. The feed intake for birds fed Diet0, Diet1, Diet2, and Diet3 was not significantly different (**Table 4**). The daily weight gain and FCR of birds fed the various diets did not vary significantly. These findings were similar to those reported by Mohammed et al. (52) and Choi et al. (53), who reported that the body weight gain and FCR of broiler chicken fed insect-based diets was not adversely affected. Mohammed et al. (52) demonstrated that the use of BSFL meal to replace FM up to 33.3% in broiler finisher diets did not significantly affect body weight gain. On the other hand, heavier body weights of birds fed dietary treatments containing BSFL meal showed no significant effect on daily weight gain and FCR in local poultry breed (54). The study results also support the recommendations by Gasco et al. (45) and Mahmud et al. (55), who advocated for the use insect meals as a suitable alternative to conventional protein resources, with no significant effect on poultry performance.

**Table 4:** Growth performance of starters and growers fed on diets containing BSFL meal.

Starter phase (Day 7 - 56)	Experimental diets							
	Diet_0_	Diet_1_	Diet_2_	Diet_3_	Diet_4_	F value	df	P Value
Initial body weight (g)	70.7 ± 1.49	69.6 ± 1.09	70.8 ± 1.24	71.7 ± 0.93	70.7 ± 0.66	0.523	4,43	0.7190
Final body weight (g)	740.1 ± 22.10^a^	524.8 ± 22.29^c^	662.9 ± 10.52^b^	581.8 ± 15.38^c^	529.3 ± 14.98^c^	26.713	4,43	0.0001
Daily weight gain (g)	13.7 ± 0.46^a^	9.3 ± 0.45^c^	12.1 ± 0.19^b^	10.4 ± 0.31^c^	9.4 ± 0.30^c^	28.522	4,43	0.0001
Daily feed intake (g/day)	37.8 ± 0.61^bc^	41.4 ± 0.98^a^	39.3 ± 0.48^ab^	39.5 ± 0.52^ab^	35.6 ± 0.95^c^	8.529	4,43	0.0001
Feed conversion ratio	2.8 ± 0.07^c^	4.5 ± 0.21^a^	3.3 ± 0.05^c^	3.8 ± 0.08^b^	3.8 ± 0.13^b^	27.413	4,43	0.0001
Grower phase (Day 56 – 126)	Diet_0_	Diet_1_	Diet_2_	Diet_3_	Diet_4_			
Initial body weight (g)	740.1 ± 22.10^a^	524.8 ± 22.29^c^	662.9 ± 10.52^b^	581.8 ± 15.38^c^	529.3 ± 14.98^c^	26.713	4,43	0.0001
Final body weight (g)	1673.6 ± 96.49	1458.1 ± 79.82	1564.7 ± 100.19	1402.6 ± 85.46	1383.0 ± 78.82	1.87	4,43	0.1330
Daily weight gain (g)	13.3 ± 1.11	13.3 ± 1.20	12.9 ± 1.35	11.7 ± 1.23	12.2 ± 1.01	0.370	4,43	0.8289
Daily feed intake (g/day)	64.76 ± 1.63^a^	66.08 ± 2.77^a^	69.988 ± 3.07^ac^	59.89 ± 1.47^ab^	52.31 ± 1.94^b^	9.171	4,43	0.0001
Feed conversion ratio	5.2 ± 0.64	5.4 ± 0.67	5.9 ± 0.64	5.9 ± 1.00	4.7 ± 0.61	0.518	4,43	0.7230
Entire phase	Diet_0_	Diet_1_	Diet_2_	Diet_3_	Diet_4_			
Initial body weight (g)	70.7 ± 1.49	69.6 ± 1.09	70.8 ± 1.24	71.7 ± 0.93	70.7 ± 0.66	0.523	4,43	0.7190
Final body weight (g)	1673.6 ± 96.49	1458.1 ± 79.82	1564.7 ± 100.19	1402.6 ± 85.46	1383.0 ± 78.82	1.87	4,43	0.1330
Daily weight gain (g)	13.5 ± 0.82	11.7 ± 0.67	12.6 ± 0.84	11.2 ± 0.72	11.0 ± 0.66	1.885	4,43	0.1301
Daily feed intake (g/day)	53.65 ± 1.08^ab^	55.94 ± 1.91^ab^	57.34 ± 1.80^a^	51.48 ± 0.84^ab^	45.44 ± 1.27^c^	10.710	4,43	0.0001
Feed conversion ratio	3.7 ± 0.10^a^	4.2 ± 049^a^	3.9 ± 0.54^a^	4.0 ± 0.48^a^	3.6 ± 0.42^a^	0.333	4,43	0.8540

Means in the same row having different superscripts are significantly different (p > 0.05).

### Economic performance

Our results revealed a significant treatment effect on the cost of feed consumed during the starter and grower chicken feeding phases (**Table 5**). Birds fed on Diet4 had the lowest cost of feed consumed at the starter and grower phases.

**Table 5:** Economic analysis on using BSFL meal in IIC diets.

Cost of feed (KES/1000g	Experimental diets							
	Diet_0_	Diet_1_	Diet_2_	Diet_3_	Diet_4_	F value	df	P Value
Starter phase	55.0875	52.2875	49.9875	46.9875	44.3875			
Grower phase	43.45	39.39	37.26	41.5	41.96			
Total feed intake (g/bird)	Diet_0_	Diet_1_	Diet_2_	Diet_3_	Diet_4_			
Starter phase	1850.68 ± 29.89^ad^	2030.52 ± 47.84^b^	1924.14 ± 23.35^ab^	1933.92 ± 25.64^ab^	1746.01 ± 46.33^acd^	8.529	4,43	0.0001
Grower phase	4533.48 ± 114.40^a^	4625.93 ± 194.03^a^	4899.23 ± 214.64^a^	4192.54 ± 102.84^ab^	3661.43 ± 135.77^b^	9.171	4,43	0.0001
Entire phase	6384.16 ± 128.58^a^	6656.46 ± 226.73^a^	6823.38 ± 213.74^ac^	6126.46 ± 99.69^a^	5407.44 ± 151.15^b^	10.710	4,43	0.0001
Cost of feed consumed (KES/bird)								
Starter phase	102.0 ± 1.64	107.2 ± 2.50	96.2 ± 1.17	90.9 ± 1.20	77.5 ± 2.06	38.573	4,43	0.0001
Grower phase	197.0 ± 4.97^a^	182.2 ± 7.64^a^	182.6 ± .8.00^a^	174.0 ± 4.27^ab^	153.6 ± 5.70^b^	6.426	4,43	0.0004
Cost of chicks (KES/bird)	100	100	100	100	100			
Total Production Cost	399.0 ± 5.79^a^	388.4 ± 9.40^ab^	378.7 ± 8.00^ab^	364.9 ± 4.14^b^	331.1 ± 6.40^c^	14.302	4,43	0.0001
Sale of birds^1^	700	700	700	700	700			
Gross Profit	301.1 ± 5.79^a^	311.6 ± 9.40^ab^	321.3 ± 8.00^ab^	335.1 ± 4.14^b^	368.9 ± 6.40^c^	14.302	4,43	0.0001
Gross profit margin	43.0 ± 0.83^a^	44.5 ± 1.34^ab^	45.9 ± 1.14^ab^	47.9 ± 0.60^b^	52.7 ± 0.91^c^	14.302	4,43	0.0001
Cost Benefit Ratio (CBR.)	1.76 ± 0.02^a^	1.81 ± 0.04^ab^	1.86 ± 0.04^ab^	1.92 ± 0.02^b^	2.12 ± 0.04^c^	16.949	4,43	0.0001
Return on Investment (RoI)	75.76 ± 2.49^a^	81.12 ± 4.07^ab^	85.51 ± 4.05^ab^	92.08 ± 2.24^b^	112.06 ± 3.81^c^	16.949	4,43	0.0001

Means in the same row having different superscripts are significantly different (p > 0.05), ^1^KES/bird.

**Figure 1:**
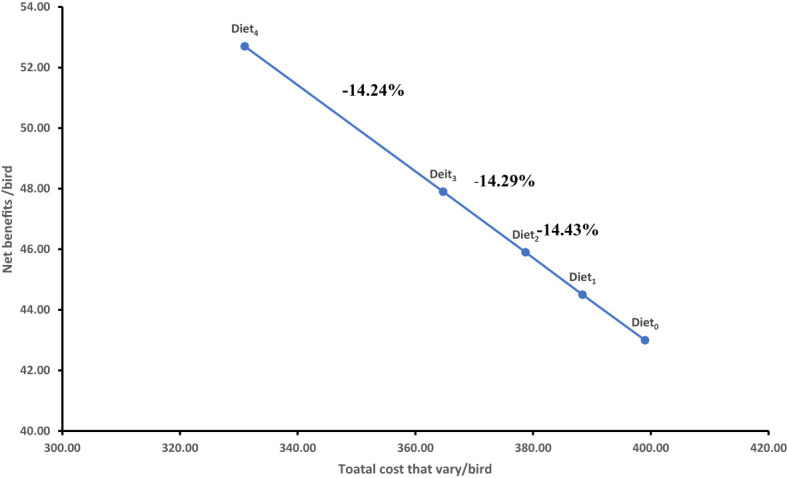
The marginal rate of return (%) of diets containing different fish-meal substitution levels with BSFL meal and conventional feeds. BSFL meal inclusion rates were: 0% (Diet_0_), 5% (Diet_1_), 10% (Diet_2_), 15% (Diet_3_), and 20% (Diet_4_) to replace fish meal.

The highest gross profit margin was recorded when birds were fed on Diet4, although this varied considerably across the different dietary treatments with insect-based meals. The CBR and RoI did not vary significantly when birds were fed on Diet0, Diet1, and Diet2. The lowest marginal rate of return (–14.24%) (**Table 6**) was recorded when farmers change from Diet3 to Diet4, which was below a minimum return of 100% (**Figure 1**). This indicated that for every US $1/bird invested, the farmer would recover US$1/bird and lose US$0.14/bird in terms of net benefits. This implies that farmers changing the feeding regime from Diet4 to Diet1 would lead to a loss of net benefits. These findings are similar to that reported by Onsongo et al. (35) who reported that the cost of feed consumed by the birds reduces with increasing inclusion levels of BSFL meal as a replacement of conventional fish-meal sources in broiler diets. Similarly, the inclusion of BSFL meal in layer starter and grower diets also demonstrated economic viability of insect-based feeds (56), which might be attributed to reduced cost of feeds with 100% substitution of the expensive fish meal with BSFL meal (35, 56). According to Chia et al. (32), there is improved economic performance with increased integration of higher inclusion levels of BSFL meals, which demonstrates sustainability and viability of BSFL meal as a promising alternative protein source to close the nutrient gap in the animal feed industry (57).

**Table 6:** Marginal and gross margin analysis (US$/bird) of insect-based feeds.

Diet type	Cost that varies US$/bird	Marginal costs	Net benefit US$/bird	Marginal net benefits	Marginal return rate (%)
Diet_4_	331.0	–	52.7	–	–
Diet_3_	364.7	33.70	47.9	-4.8	-14.24
Diet_2_	378.7	14.0	45.9	-2	-14.29
Diet_1_	388.4	9.7	44.5	-1.4	-14.43
Diet_0_	399.0	–	43	–	–

## Conclusion

Overall, the present study has provided new data and knowledge on the potential use of a new sustainable feedstuff for improved indigenous chickens. The main findings of the current research suggest that full-fat BSFL meal can be used to up to 20% level of inclusion in starter and grower chicken diets, without detrimental effects on growth performance. Remarkable differences in the feed intake and FCR were found in relation to the different diet types with varying nutritional profiles. On this background, BSFL meal can be considered as a suitable and affordable alternative protein feed resource for IIC diets. However, important efforts should be made to evaluate new processing techniques such as defatting of the BSFL meal, which is capable of improving the protein profile of larvae, thus potentially counteracting the negative effects on the nutritional value, perceived healthiness, and economic benefits of the poultry meat. Defatted BSFL meal will also reduce the risk of lipid oxidation, allowing for a longer shelf life and proper mixing of the product for optimal feed formulation. These defatted BSFL meal or oils should also be evaluated from a safety point of view, as new evidence on their safety could be adopted to reduce the potential toxicity of the meal.

## Data availability statement

The original contributions presented in the study are included in the article/supplementary material. Further inquiries can be directed to the corresponding author.

## Ethics statement

This study was reviewed and approved by Ethical approval for the study was provided by the Institutional Animal Care and Use Committee (IACUC) of Kenya Agricultural and Livestock Research Organization (KALRO)-Veterinary Science Research Institute (VSRI); approval Code No.: KALROVSRI/IACUC019/30082019. Written informed consent was obtained from the owners for the participation of their animals in this study.

## Author contributions

Conceptualization, MW, IO, LK, AW, and CT. Methodology, MW, IO, LK, and CT. Software, IO. Validation, MW, IO, LK, AW, and CT. Formal analysis, MW, IO, LK, AW, and CT. Investigation, MW, IO, LK, AW, and CT. Resources, CT. Data curation, MW, IO, LK, AW, and CT. Writing—original draft preparation, MW, IO, BM, SS, LK, AW, and CT. Writing—review and editing, MW, IO, LK, AW, and CT. Visualization, MW, IO, LK, AW, and CT. Supervision, IO, LK, and AW. Project administration, CT. Funding acquisition, CT. All authors contributed to the article and approved the submitted version.

## Funding

The authors gratefully acknowledge the financial support from the Australian Centre for International Agricultural Research (ACIAR) (ProteinAfrica – Grant No: LS/2020/154), the Rockefeller Foundation (WAVE-IN—Grant No.: 2021 FOD 030), Norwegian Agency for Development Cooperation, the Section for research, innovation, and higher education grant number (Grant No.: RAF–3058 KEN–18/0005) (CAP–Africa), the Curt Bergfors Foundation Food Planet Prize Award, the Swedish International Development Cooperation Agency (Sida); the Swiss Agency for Development and Cooperation (SDC); the Federal Democratic Republic of Ethiopia; and the Government of the Republic of Kenya. The funders had no role in the study design, data collection, and analysis, decision to publish, or preparation of the manuscript. Therefore, the views expressed herein do not necessarily reflect the official opinion of the donors.

## Acknowledgments

The support and commitment from the technical staff of icipe and KALRO, Naivasha, is highly appreciated.

## Conflict of interest

The authors declare that the research was conducted in the absence of any commercial or financial relationships that could be construed as a potential conflict of interest.

## Publisher’s note

All claims expressed in this article are solely those of the authors and do not necessarily represent those of their affiliated organizations, or those of the publisher, the editors and the reviewers. Any product that may be evaluated in this article, or claim that may be made by its manufacturer, is not guaranteed or endorsed by the publisher.
